# Patients with Acute Myeloid Leukemia Admitted to Intensive Care Units: Outcome Analysis and Risk Prediction

**DOI:** 10.1371/journal.pone.0160871

**Published:** 2016-08-30

**Authors:** Michele Pohlen, Nils H. Thoennissen, Jan Braess, Johannes Thudium, Christoph Schmid, Matthias Kochanek, Karl-Anton Kreuzer, Pia Lebiedz, Dennis Görlich, Hans U. Gerth, Christian Rohde, Torsten Kessler, Carsten Müller-Tidow, Matthias Stelljes, Thomas Büchner, Günter Schlimok, Michael Hallek, Johannes Waltenberger, Wolfgang Hiddemann, Wolfgang E. Berdel, Bernhard Heilmeier, Utz Krug

**Affiliations:** 1 Department of Medicine A, Hematology and Oncology, University Hospital Muenster, Muenster, Germany; 2 Department of Medicine III, Hematology and Oncology, University Hospital Grosshadern, Munich, Germany; 3 Department of Medicine II, Klinikum Augsburg, Augsburg, Germany; 4 Department of Medicine I, University Hospital Cologne, Cologne, Germany; 5 Department of Cardiovascular Medicine, University Hospital Muenster, Muenster, Germany; 6 Institute of Biostatistics and Clinical Research, University Muenster, Muenster, Germany; 7 Department of Medicine D, Hematology and Oncology, University Hospital Muenster, Muenster, Germany; Queen's University Belfast, UNITED KINGDOM

## Abstract

**Background:**

This retrospective, multicenter study aimed to reveal risk predictors for mortality in the intensive care unit (ICU) as well as survival after ICU discharge in patients with acute myeloid leukemia (AML) requiring treatment in the ICU.

**Methods and Results:**

Multivariate analysis of data for 187 adults with AML treated in the ICU in one institution revealed the following as independent prognostic factors for death in the ICU: arterial oxygen partial pressure below 72 mmHg, active AML and systemic inflammatory response syndrome upon ICU admission, and need for hemodialysis and mechanical ventilation in the ICU. Based on these variables, we developed an ICU mortality score and validated the score in an independent cohort of 264 patients treated in the ICU in three additional tertiary hospitals. Compared with the Simplified Acute Physiology Score (SAPS) II, the Logistic Organ Dysfunction (LOD) score, and the Sequential Organ Failure Assessment (SOFA) score, our score yielded a better prediction of ICU mortality in the receiver operator characteristics (ROC) analysis (AUC = 0.913 vs. AUC = 0.710 [SAPS II], AUC = 0.708 [LOD], and 0.770 [SOFA] in the training cohort; AUC = 0.841 for the developed score vs. AUC = 0.730 [SAPSII], AUC = 0.773 [LOD], and 0.783 [SOFA] in the validation cohort). Factors predicting decreased survival after ICU discharge were as follows: relapse or refractory disease, previous allogeneic stem cell transplantation, time between hospital admission and ICU admission, time spent in ICU, impaired diuresis, Glasgow Coma Scale <8 and hematocrit of ≥25% at ICU admission. Based on these factors, an ICU survival score was created and used for risk stratification into three risk groups. This stratification discriminated distinct survival rates after ICU discharge.

**Conclusions:**

Our data emphasize that although individual risks differ widely depending on the patient and disease status, a substantial portion of critically ill patients with AML benefit from intensive care.

## Introduction

AML is the most common type of acute leukemia in adults, accounting for approximately 2.8% of all cancer worldwide [1]. Without treatment, AML is fatal, but therapy and prognosis have improved in recent decades [2]. Intensive chemotherapy, either alone or followed by autologous or allogeneic stem cell transplantation, has the potential to cure AML, and long-term survival of patients is increasing [3]. However, such intensive treatment is associated with potentially life-threatening toxicities, particularly in elderly patients. Although decision tools might aid decisions regarding which elderly patients are amenable to intensive treatment [4,5], approximately 13% of patients with AML require ICU treatment [6]. Several studies have described clinical outcomes and prognostic factors for patients with or without other hematological malignancies. The majority of these studies are of limited value because they were based on small cohorts, not validated in an independent cohort, included unselected patients with solid cancer and hematological malignancies, did not distinguish between ICU and hospital mortality, did not analyze survival and/or were solely focused on complications [6–16].

The decision to admit a patient to the ICU is often an ethical dilemma and is based on the individual clinician’s decision, which is loosely based on established scores (e.g., SOFA, LOD, APACHE II, SAPS II) [17–20]. Because these scores are based on and designed for analysis of unselected patients admitted to the ICU, patients with cancer, particularly those with AML, are underrepresented. For example, APACHE II and SAPS II consider malignancy as a risk factor without further differentiation of the type or disease status of the malignancy. APACHE II considers only five points for immunocompromised, non-operative patients, while allowing a total range between 0 and 71.

The objective of this study was to establish and validate risk factors associated with mortality during and after ICU treatment based on a large and multicenter cohort and to establish potential risk scores.

## Materials and Methods

### Patients and treatment

The AML in ICU score was established in a cohort of 187 adults with AML admitted to the ICU at the University Hospital of Muenster, representing all patients with a diagnosis of AML admitted to the ICU between 11/2004 and 09/2011. Data were collected retrospectively from patient records and follow-up physicians. Prior to analysis, the data were anonymized and de-identified. Approval for this investigation was obtained from the Ethics Board of Westfalian Wilhelms-University Muenster and the Physicians Chamber of Westfalia-Lippe, Germany (2015-688-f-S).

Validation was performed on a cohort of 264 patients with AML admitted to the ICU at the University Hospital of Grosshadern in Munich, the University Hospital of Cologne and the Municipal Hospital of Augsburg (all located in Germany) between 01/2004 and 02/2010.

Intensive induction treatment consisted of any of the following: “7+3” (cytarabine 100 mg/m² once daily on days 1–7 as a 24-h intravenous infusion and daunorubicin 45 mg/m² once daily on days 3–5, intravenous infusion; “7+GO” (cytarabine 100 mg/m² once daily on days 1–7 as a 24-h intravenous infusion and gemtuzumab ozogamicin 6 mg/m² once on day 1 and 4 mg/m² once on day 8, intravenous infusion); “TAD” (tioguanine 100 mg/m² twice per day on days 3–9, orally; cytarabine 100 mg/m² on days 1–2 as an intravenous infusion and 100 mg/m² twice daily on days 3–8, intravenous infusion; and daunorubicin 60 mg/m² once daily on days 3–5, intravenous infusion); “HAM” (high-dose cytarabine 3 g/m² twice daily on days 1–3, intravenous infusion, and mitoxantrone 10 mg/m² once daily on days 3–5, intravenous infusion; patients >60 years of age received only 1g/m² cytarabine); and “S-HAM” (high-dose cytarabine 3g/m² twice daily on days 1–2 and 8–9, intravenous infusion, and mitoxantrone 10 mg/m² once daily on days 3–4 and 10–11, intravenous infusion; patients >60 years of age received 1 g/m² cytarabine). With the exception of dose-dense S-HAM induction, patients <60 years of age routinely received two induction courses, whereas patients aged 60+ received a second induction course only in case of persisting bone marrow blasts on day 15 after the start of treatment. Postremission treatment consisted of high-dose cytarabine in case of “7+3” or “7+GO” induction (patients <60 years received three courses of cytarabine 3g/m² twice daily on days 1, 3 and 5, intravenous infusion; those 60 years or older only received two courses of cytarabine 3g/m² twice daily on days 1, 3 and 5, intravenous infusion). Postremission treatment also consisted of TAD consolidation followed by prolonged monthly maintenance therapy in case of “TAD(-HAM)”, “HAM(-HAM)” or “S-HAM” induction or was followed by autologous SCT in younger patients. Details of the treatment protocols have been published elsewhere [21–23]. According to the patient’s risk of relapse, allogeneic stem cell transplantation was performed alternatively to the scheduled postremission treatment in patients in first remission or after relapse when possible. Conditioning protocols varied and depended on the remission status and age of the patients [24–26].

Only disease status was included in the analysis. Owing to the broad heterogeneity of the applied protocols, a detailed analysis of chemotherapy regimen related to death in the ICU and survival after ICU discharge could not reasonably be performed. (Re)induction protocols are applied in active disease or at primary diagnosis, and consolidation regimens are administered in cases of complete remission. Owing to this strong correlation, information about chemotherapy protocols is already included in the variable disease status.

### Endpoints

Complete remission was defined as hematological recovery with at least 1,000 neutrophils per μl, at least 100,000 platelets per μl, and < 5% bone marrow blasts. ICU mortality was defined as death at any time during the course of the ICU stay. Overall survival after ICU discharge was survival from the day of ICU discharge until death from any cause, and censoring of patients known to be alive at the time of last follow-up was performed. The term mortality, when given as a percentage, was defined as the number of deaths per number of ICU stays observed.

### Definition of variables

Arterial oxygen partial pressure (paO2) was evaluated at the time of ICU admission, irrespective of oxygen supply. Active AML at the time of ICU admission included 1, patients with primary diagnosed AML; 2, patients with relapsed AML before or during reinduction therapy or before evaluation of the remission status at the time of ICU admission; and 3, patients with persisting disease after induction or reinduction therapy at the last evaluation of disease status preceding ICU admission. Advanced AML at the time of ICU discharge was defined as refractory or relapsed AML status. Serve infections as reason for ICU admission required at least two of the following criteria: temperature >38°C (100.4°F) or <36°C (96.8°F), tachycardia (>90 bpm), tachypnea >20/min or clinically proved infectious complications (like microbiologically documented infection or radiographic signs of an infection). To avoid affections by the AML and/or associated therapy, leukocyte count was not considered. Days until ICU admission were the days between hospital admission and ICU admission. The Glasgow Coma Scale (GCS) was applied as previously described [27]. The Simplified Acute Physiology Score (SAPS) II was routinely determined in all ICU patients [28]. Additionally, the LOD and SOFA score were calculated in the training cohort to enhance comparability to other ICU scores [19,20,29]. Variables used for calculation of all scores were determined at the time of admission. As prothrombin values were not available in most cases and to avoid potential bias, LOD was thoroughly calculated without prothrombin values. Cytogenetic and molecular genetic risk were classified according to European LeukemiaNet (ELN) guidelines 2010 [30].

### Statistical Analysis

Correlations with ICU mortality were evaluated using the Chi-square test for categorical data and the Mann-Whitney U test for continuous variables. Only variables with less than 10% missing values in the training cohort were considered. Missing values were replaced by the median value of the variable. Continuous variables were categorized where appropriate (S1 Table). Variables with p<0.1 in the univariate evaluation were selected for a multivariate binary logistic regression with stepwise backward selection and a threshold value of 0.05 for inclusion and 0.1 for exclusion. The final model of the variable selection process was used as the scoring model.

Survival analyses were performed by Kaplan Meier estimates. Correlations with survival were evaluated by the log-rank test; parameters with p<0.1 in the log-rank test were evaluated in multivariate Cox regression analysis with stepwise backward selection and a threshold value of 0.05 for inclusion and 0.1 for exclusion. Unless otherwise stated, the significance level was alpha = 0.05 in all analyses.

All statistical analyses were performed in cooperation with the Institute of Biostatistics and Clinical Research of the University Hospital of Muenster, Germany, and were computed with IBM SPSS Statistics for Windows, Version 22.0 (IBM Corp., Armonk, NY).

## Results

### Patient characteristics

Patient characteristics are presented in Table 1. At the time of ICU admission, the median age of the patients was 59 years. Compared with the training cohort, the validation cohort included more patients with newly diagnosed AML and fewer patients in remission. The training cohort also had lower paO2 and hematocrit at the time of ICU admission. Among patients surviving the ICU, the median duration of treatment was three days in the validation cohort and four days in the training cohort. Age and sex, the combined cytogenetic and molecular risk profile according to ELN2010 classification, the proportion of patients who had previously undergone allogeneic stem cell transplantation, the reason for ICU admission, and the proportion of patients requiring mechanical ventilation or dialysis in the ICU were distributed similarly between the training and validation cohorts (Table 1). Information about type of AML (*de novo* versus secondary), time interval between hospital admission and ICU admission, mean arterial pressure at ICU admission, diuresis and GCS at the time of ICU admission were not available for the validation cohort.

**Table 1:** Baseline characteristics of all patients and patients surviving the ICU stay in the training and validation cohorts.

Parameter	All patients			ICU survivors		
	Training cohort	Validation cohort	p	Training cohort	Validation cohort	p
No of patients / no of ICU stays	187 / 187	264 / 363		79 / 79	175 / 232	
Age in years, median (range)	59 (16–83)	58 (17–85)	0.335	58 (16–83)	58 (20–85)	0.797
Male sex, n (%)	112 (60)	141 (53)	0.172	42 (53)	88 (50)	0.671
Type of AML, n (%)						
de novo	136 (73)	n.a.		58 (73)	n.a.	
Secondary AML	51 (27)	n.a.		21 (27)	n.a.	
ELN 2010 risk classification, n (%)			0.796			0.968
Low risk	33 (18)	45 (21)		19 (24)	33 (22)	
Intermediate-I	81 (43)	91 (43)		30 (38)	61 (41)	
Intermediate-II	17 (9)	18 (9)		7 (9)	14 (9)	
High risk	56 (30)	57 (27)		23 (29)	41 (28)	
Disease status at ICU admission, n (%)			**<0.001**			**<0.001**
Newly diagnosed / not yet evaluated	78 (42)	194 (53)		26 (33)	136 (59)	
In remission	63 (34)	68 (19)		33 (42)	37 (16)	
Relapsed or refractory	46 (25)	101 (28)		20 (25)	59 (25)	
Previous allogeneic SCT, n (%)	56 (30)	98 (27)	0.466	20 (25)	48 (21)	0.390
Reason for ICU admission, n (%)			0.189			**0.047**
Severe infection	95 (51)	163 (45)		19 (24)	84 (36)	
Temperature > 38°C (100.4°F) or < 36°C (96.8°F)	63 (66)	81 (49)		9 (47)	39 (46)	
Tachycardia (> 90 bpm)	80 (84)	127 (77)		12 (63)	53 (60)	
Tachypnea > 20 /min	67 (71)	93 (57)		9 (47)	49 (58)	
Microbiological findings	40 (42)	69 (42)		7 (36)	29 (34)	
other reasons	92 (49)	200 (55)		60 (76)	148 (64)	
Time between hospital admission and ICU admission in days, median (range)	12 (0–90)	n.a.		14 (0–43)	n.a.	
Time spent in ICU in days, median (range)	n.e.	n.e.		4 (0–65)	3 (0–66)	**0.021**
paO2 in mmHg, median (range)	76.5 (32–217)	82 (40–426)	**0.042**	80 (41.5–160)	84 (40–426)	0.502
Mean arterial pressure (MAP) in mmHg, median (range)	85 (40–155)	n.a.		85 (40–130)		
Hematocrit in %, median (range)	25 (13–44)	27 (18–44)	**<0.001**	24 (13–43)	28 (18–44)	**<0.001**
Urine production in l/24h, median (range)	1.45 (0–8.5)	n.a.		1.9 (0.1–8.4)	n.a.	
Glasgow Coma Scale (GCS), median (range)	15 (3–15)	n.a.		10 (3–15)	n.a.	
Patients with invasive ventilation, n (%)	110 (59)	179 (53)	0.162	24 (30)	63 (29)	0.858
Patients with hemodialysis on ICU, n (%)	58 (31)	88 (25)	0.120	8 (10)	29 (9)	0.828

Comparisons (Mann-Whitney U test for continuous variables and Chi-square test for categorized variables) were performed between the learning and validation cohorts. Patient-specific variables (age, sex, ELN2010 risk classification) were calculated according to the number of patients in the validation cohort, whereas situation-specific variables (disease status, previous allogeneic transplantation, reason for ICU admission, duration of ICU stay, paO2 at ICU admission, hematocrit at ICU admission, mechanical ventilation and hemodialysis on ICU) were calculated according to ICU stays.

Abbreviations: ELN, European LeukemiaNet; n.a., not available; n.e., not evaluated; paO2, arterial oxygen partial pressure; SCT, stem cell transplantation

### Prognostic factors influencing ICU mortality

An overview of all parameters selected for analysis and their classification is presented in S1 Table.

**Fig 1:**
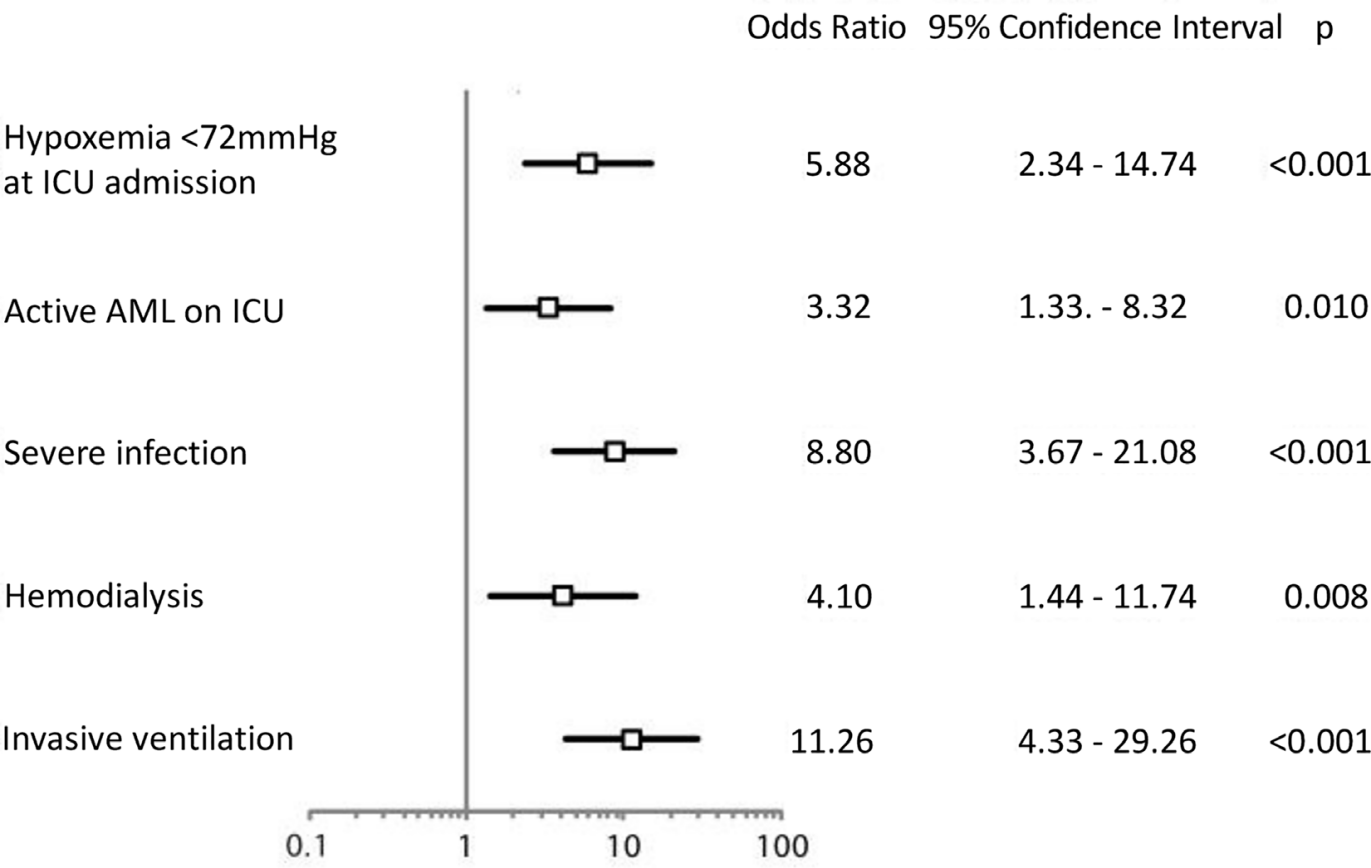
Odds ratio (OR) plot of parameters associated with mortality in the ICU (intensive care unit). Abbreviations: paO2, arterial oxygen partial pressure <72 mmHg at ICU admission.

Independent prognostic factors for ICU mortality in a multivariate model, displayed in Fig 1, were as follows: paO2 <72 mmHg at ICU admission, active AML in the ICU (relapsed, refractory, newly diagnosed), severe infection at ICU admission, and need for hemodialysis and mechanical ventilation. On the basis of these five variables, a logistic model was generated for the prediction of mortality in the ICU. The fitted logistic model is described by formulas 1 and 2, which can be used to calculate the predicted ICU mortality of each patient.

Formula 1:

X=-3.921+(1.771xhypoxemia)+(1.200xactive AML on ICU)+(2.175xsevere infection)+(1.412xhemodialysis)+(2.421xmechanical ventilation)

Formula 2:

Predicted ICU mortality=1/[1+exp(-X)]

Coding of parameters used in formula 1:

Hypoxemia: paO2 <72 mmHg at admission = 1; paO2 ≥72 mmHg at admission = 0

Active AML on ICU: relapsed, refractory, newly diagnosed AML = 1; remission = 0

Severe infection: reason for ICU admission was infection with two of the following criteria (temperature > 38°C or < 36°C, tachycardia > 90 bpm, or tachypnea > 20 /min) = 1; other reason for ICU admission = 0

Hemodialysis: need for hemodialysis = 1; no need for hemodialysis = 0

Mechanical ventilation: need for mechanical ventilation = 1; no need for mechanical ventilation = 0

**Fig 2:**
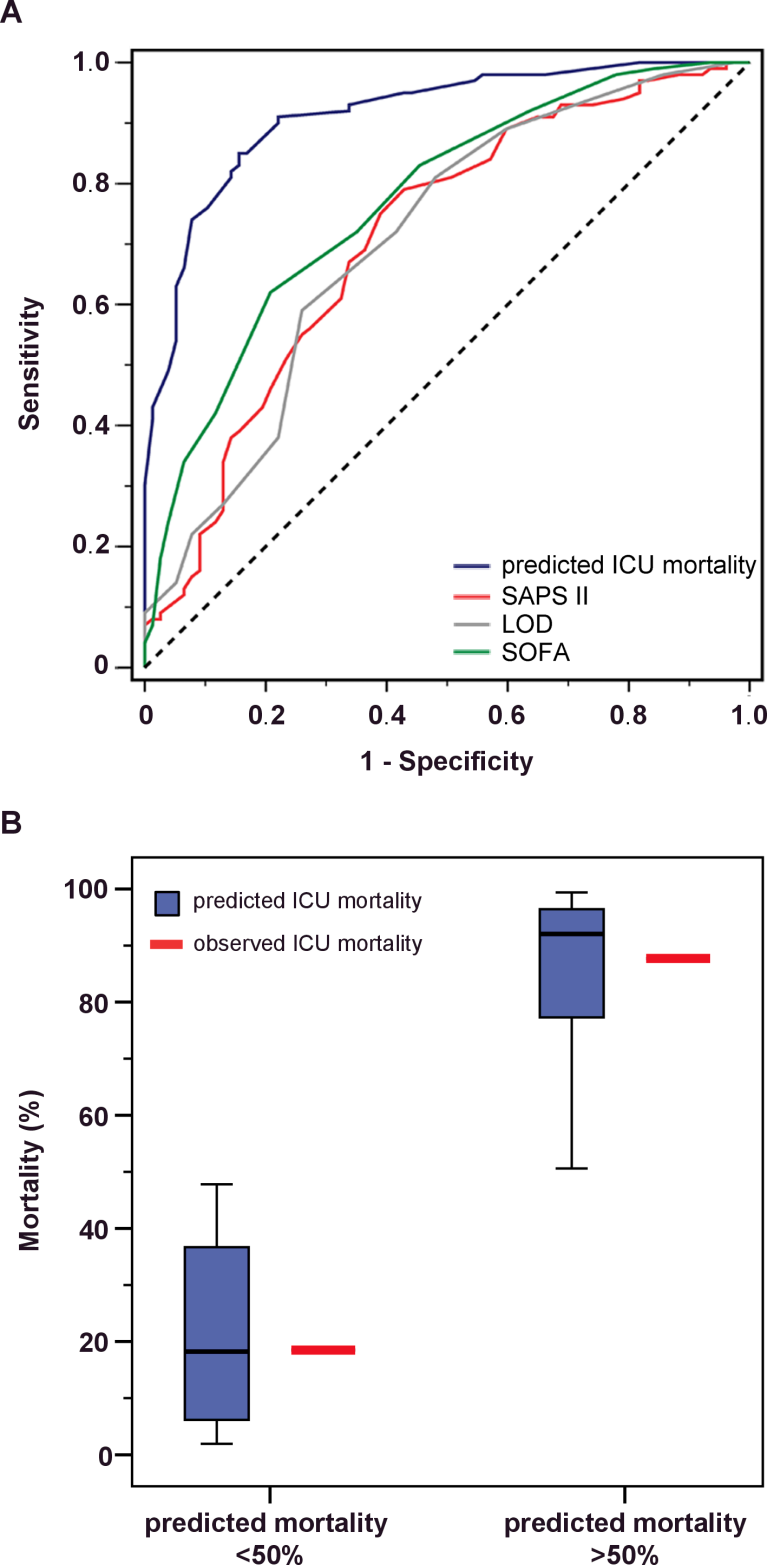
Correlation of predicted versus actual ICU mortality (intensive care unit) in the training cohort. (A) Receiver operator characteristics for the different scores with the area under the curve (AUC). Score 1: novel mortality score. Score 2: SAPS II. Score 3: LOD. Score 4: SOFA. (B) Predicted versus actual ICU mortality. Patients were classified according to their individual predicted ICU mortality (below versus ≥50%; boxes represent the interquartile range (IQR); whiskers indicate the minimum and maximum values but are not longer than 1.5 times the length of the corresponding box; values outside this range are represented by separate dots), which is plotted against the actual mortality rate for the three groups.

Fig 2 shows the goodness of fit of the predicted ICU mortality as well as the SAPS II, the LOD, and the SOFA score compared with the observed ICU mortality. Fig 2A presents the receiver operator characteristics (ROC) analysis, and Fig 2B depicts a plot of the predicted versus observed mortality rates. The predicted ICU mortality had an AUC value of 0.913 (95% CI: 0.873–0.954), compared with an AUC of 0.721 (95% CI: 0.646–0.796) for the SAPSII Score in the ROC of the training cohort (Fig 2A). The AUC analysis of the LOD and the SOFA score showed similar values compares to the SAPS II Score (AUC = 0.708 [LOD] and 0.770 [SOFA]). In patients with a predicted ICU mortality of <50% (median 18%, range 2–48%), 19% (15 of 81) died in the ICU (Fig 2B). By contrast, an ICU mortality of 88% (93 of 106) was observed in the patients with a predicted ICU mortality of >50% (median 92%, range 51 to 99%) (Fig 2B).

**Fig 3:**
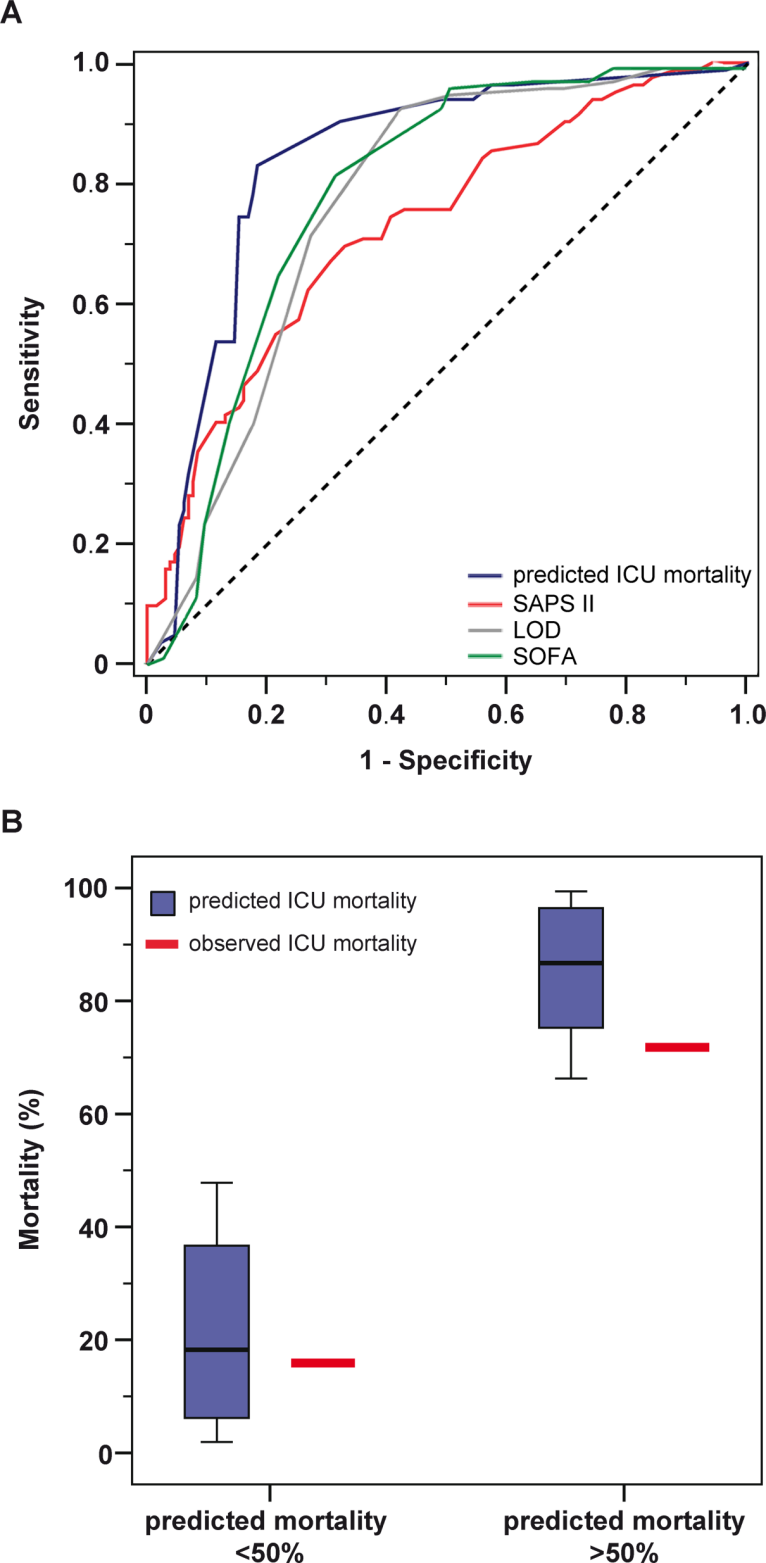
Correlation of predicted versus actual ICU mortality (intensive care unit) in the validation cohort. (A) Receiver operator characteristics for the different scores with the area under the curve (AUC). Score 1: novel score. Score 2: SAPS II. Score 3: LOD. Score 4: SOFA. (B) Predicted versus actual ICU mortality. Patients were classified according to their individual predicted mortality in the ICU (below versus ≥50%; boxes represent the IQR; whiskers indicate the minimum and maximum values, but are not longer than 1.5 times the length of the corresponding box; values outside this range are represented by separate dots), which is plotted against the actual mortality rate.

When applying our score to the validation group, predicted ICU mortality had an AUC in the ROC of 0.841 (95% CI: 0.784–0.897), compared with an AUC of 0.730 (95% CI: 0.661–0.799) for the SAPSII Score, an AUC of 0.773 (95% CI: 0.696–0.851) for the LOD score, and 0.783 for the SOFA score (95% CI: 0.714–0.864)(Fig 3A). In quantitative terms, 16% (37 of 232) of patients with a predicted ICU mortality of <50% (median 18%, range 2–48%) died in the ICU (Fig 3B). Consistent with the findings in the training cohort, the patients with a predicted ICU mortality of >50% (median 87%, range 66–99%) exhibited an increased mortality rate of 72% (94 of 131) in the ICU (Fig 3B).

### Prognostic factors for survival after ICU discharge

**Fig 4:**
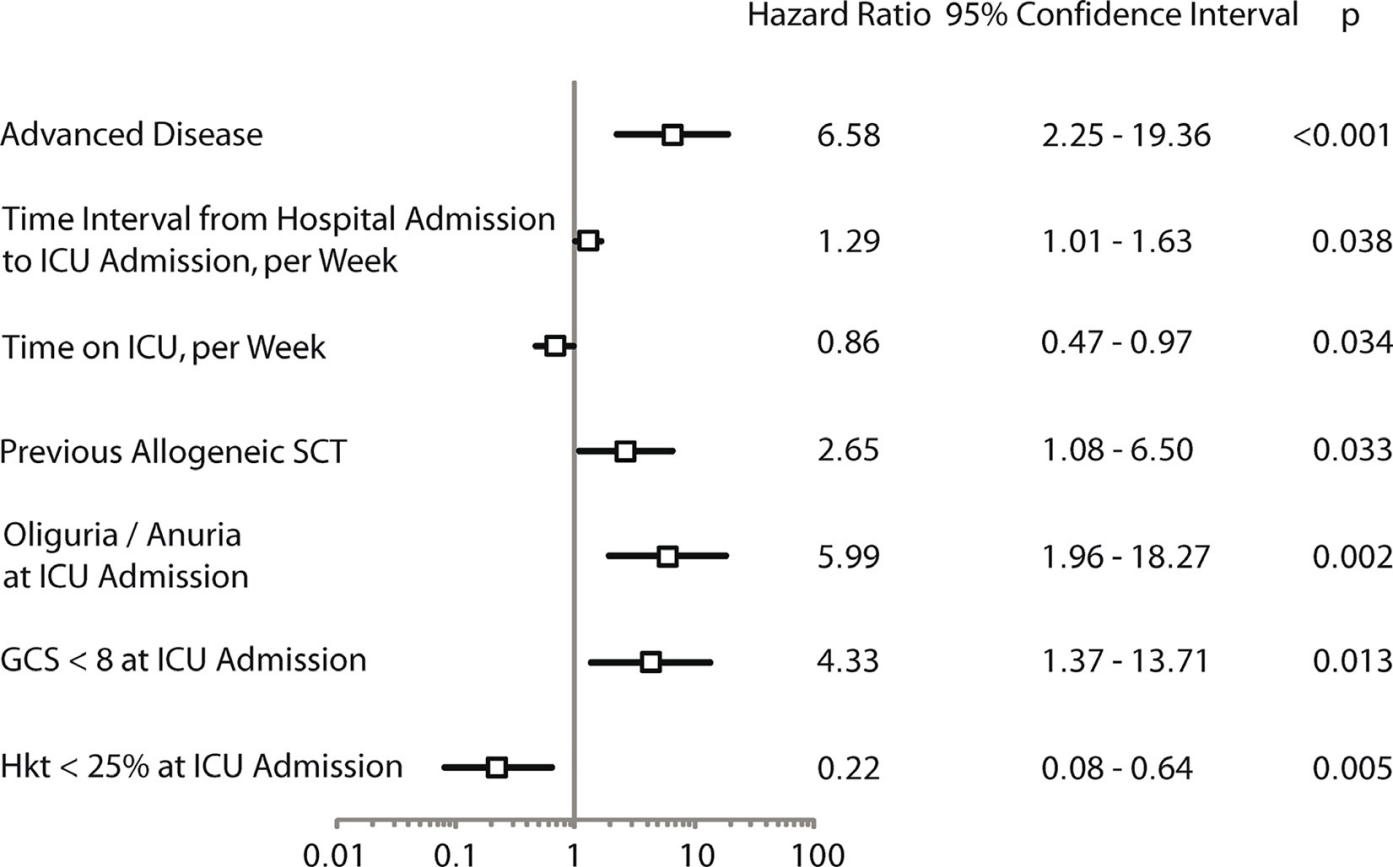
Hazard ratio (HR) plot of parameters associated with survival after ICU (intensive care unit) discharge. Abbreviations: SCT, stem cell/bone marrow transplantation; GCS, Glasgow Coma Scale; Hkt, hematocrit.

Seventy-nine patients (42%) in the training cohort survived their ICU stay. Table 1 displays the characteristics of these ICU survivors. The projected 3-year survival of this cohort from the time of ICU discharge was 64% (95% CI: 51–77%) after a median follow-up of 1.6 years. The parameters selected for the analysis of association with prognosis after ICU discharge are also listed in S1 Table. In multivariate Cox regression analysis, the following parameters were identified as independent prognostic factors for decreased survival after ICU discharge (Fig 4): advanced disease (relapsed or refractory); previous allogeneic stem cell transplantation (alloSCT); fewer days between hospital admission and ICU admission; more days spent in the ICU; impaired diuresis <1,000 ml/24 hours at ICU admission; GCS <8 at ICU admission; and a hematocrit of ≥25% at ICU admission. Based on the Cox regression model, the risk score of each patient was calculated using formula 2 and formula 3:

Formula 3:

X=(1.886xadvanced disease)+(0.974xalloSCT)+(0.036xdays in hospital before ICU admission)-(0.055xdays spent in ICU)+(1.789xdecreased urine production)+(1.465xdecreased GCS)-(1.510xdecreased hematocrit)

Coding of parameters used in formula 3:

Advanced disease: relapsed or refractory = 1; newly diagnosed or in remission = 0

alloSCT: Previous alloSCT = 1; no history of alloSCT = 0

Days in hospital before ICU admission: Number of days between hospital admission and ICU admission

Days spent in ICU: Number of days between ICU admission and ICU discharge

Decreased urine production: urine production <1,000 ml/24 hours at ICU admission = 1; urine production >1,000 ml/24 hours at ICU admission = 0

Decreased GCS: GCS <8 at ICU admission = 1; GCS ≥8 at ICU admission = 0

Decreased hematocrit: hematocrit of <25% at ICU admission = 1; hematocrit of ≥25% at ICU admission = 0

**Fig 5:**
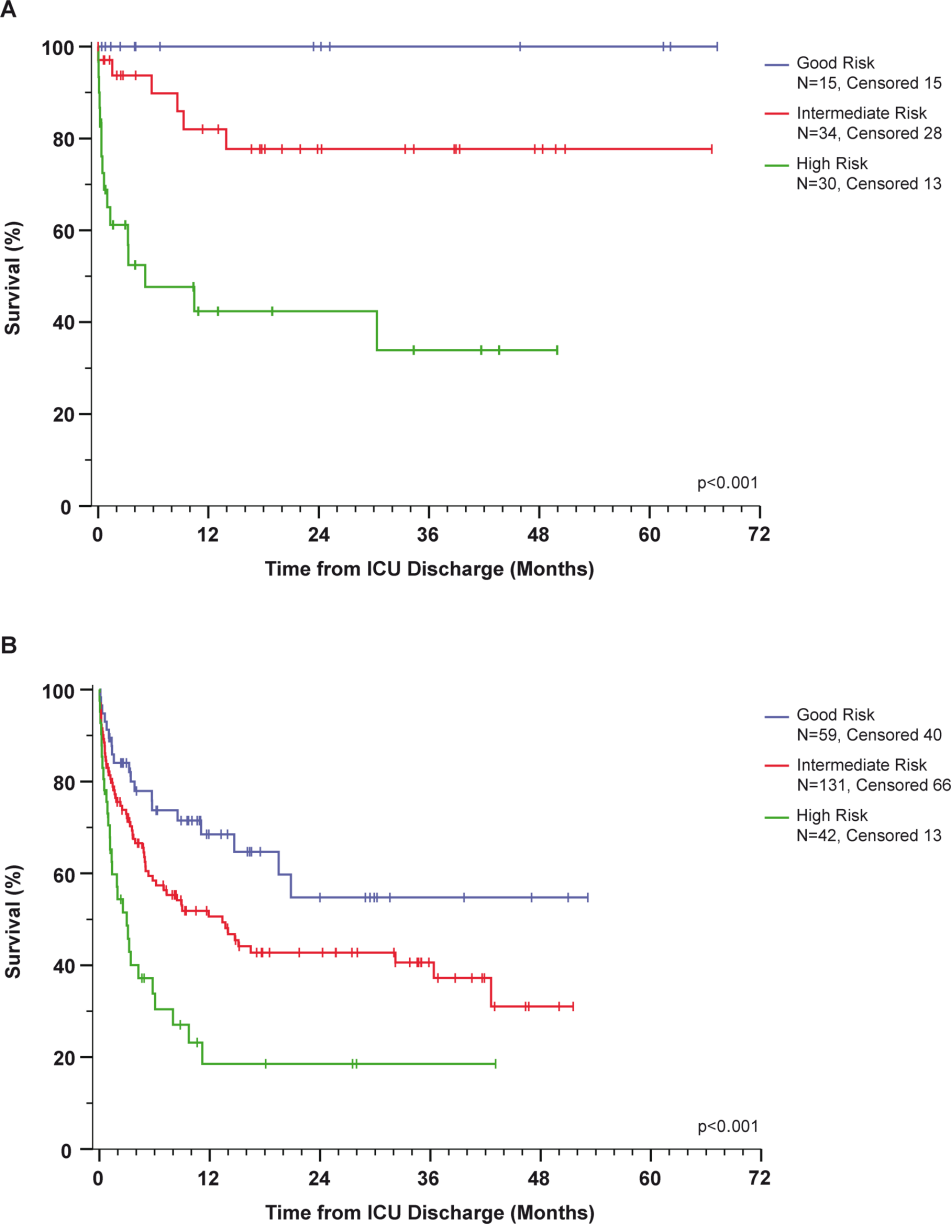
Correlation of predicted survival rate after ICU (intensive care unit) discharge with overall survival. **Patients were grouped according to their probability of survival and the corresponding Kaplan-Meier estimates.** (A) Overall survival for patients in the training cohort. (B) Overall survival for patients in the validation group.

Stratifying the ICU survivors into three risk groups according to the risk score calculated using formula 3 revealed marked differences in survival after ICU discharge. Patients with the lowest risk (X values <0.23, n = 15) displayed one-year survival after ICU discharge of 100%. Patients with intermediate risk (X values between 0.23 and 2.33, n = 34) exhibited 1-year survival of 82% (95% CI: 68–97%), and patients with the highest risk (X value >2.34, n = 30) exhibited 1-year survival of 42% (95% CI: 22–63%) (Fig 5A).

Applying the same stratification to the validation group (median follow-up of 1.4 years) revealed comparable differences in survival from the time of ICU discharge, although the survival rates were lower. One-year survival after ICU discharge was 69% (95% CI: 55–81%) in the 59 patients with the lowest risk, 51% (95% CI: 41–60%) in the group of 131 patients with intermediate risk, and 19% (95% CI: 4–33%) in the 42 patients with high risk (Fig 5B).

## Discussion

Despite encouraging survival rates of ICU survivors compared to non-ICU patients [31], assumed high mortality represents a major reason for the widespread hesitation to refer AML patients for treatment in the ICU. In addition, outcome prediction instruments are not valid in individual patients, and ICU scoring systems are only capable of describing the severity of illness of ICU cohorts. The two most commonly used scores, SAPS II and APACHE II, were established based on large numbers of unselected patients [18,28]. Because AML is rare in ICU patients, patients with AML were clearly underrepresented in the establishment of these scores, and both disease status and the impact of AML-specific procedures (such as an allogeneic stem cell transplantation) were not considered in the design of global scoring systems, thus limiting their applicability to patients with AML.

Sculier *et al*. published a report stating that neither SAPS II nor APACHE II are sufficiently accurate to be used in the routine management of cancer patients requiring ICU treatment [32]. They evaluated the prognostic value of these two scores for mortality both during the hospital stay and after discharge in 261 cancer patients admitted to the ICU. No major difference was observed between the two scoring systems, but outcome could not reliably be predicted. Subgroup analyses of patients with hematological malignancies or patients with AML were not performed in this study.

Based on the data for 451 patients with AML receiving available intensive care, the largest cohort of AML patients analyzed to date, we were able to specifically analyze prognosis in this defined patient population. Several risk predictors for ICU outcome as well as subsequent survival were identified, and we established a score predicting ICU mortality in patients with AML. This score outperformed the established SAPS II, LOD, and SOFA scores in the training and the validation cohort with respect to the area under the curve in the ROC analysis, regardless of hospital, treating physician, or treatment. Although the potential to discriminate was higher in the training cohort it was still evident in the validation cohort. The ability of our score to accurately predict ICU mortality in this independent cohort supports the reliability of the score. The results for patients with a low mortality risk may encourage clinicians to initiate or extend intensive care to AML patients. However, the decision to pursue ICU treatment for an AML patient requires an interdisciplinary approach that includes hematologists, intensive care physicians, and consideration of the patients’ wishes and expectations. Thus, such a decision can never be based solely on the results of a score.

Originally all three scores, the SAPS II, LOD, and SOFA score, were generated on the basis of the “worst” values in the first 24-hour period after admission[20,28,29]. To analyze their applicability as a prognostic tool for the clinician, all scores in this manuscript are based on data collected at the time of admission. This is an important difference to the preexisting scores and underlines the relevance of the developed scores as prognostic tool in clinical use.

Previous studies have defined single parameters predicting ICU or hospital mortality in cohorts including or comprising patients with AML, such as use of mechanical ventilation, low fibrinogen [31], use of vasopressors [8,9], increased creatinine, number of failing organ systems [8], illness severity [9], mechanical ventilation [13], sepsis, and length of hospital stay prior to ICU admission [10]. Most of these factors were verified independently by our risk factor analysis.

The mortality rate in the ICU was 58% in the training cohort and 36% in the validation cohort. The reason for this discrepancy is unclear because the differences in the baseline characteristics (Table 1) were not sufficient to provide an explanation. Admission criteria for ICU patients vary from hospital to hospital. Nevertheless, these findings are comparable to recent studies reporting mortality rates of 28–84% [6–9,15,31,33]. However, direct comparison with published mortality rates is complicated by the use of different parameters: death in ICU, death in hospital, or death after 90 days and/or one year.

In addition to predictors of ICU mortality, we also identified prognostic factors for ICU survival by AML patients. Not surprisingly, advanced disease status was a strong negative prognostic factor for survival, and impaired immune responses to pathogens, particularly in the early phase after allogeneic SCT, and severe acute and extensive chronic graft versus host disease are clearly associated with infectious complications [34].

Days spent in the hospital before ICU admission negatively influenced outcomes after ICU discharge, whereas days spent in the ICU before ICU discharge had an opposite prognostic influence on future survival. Azoulay *et al*. reported that fewer days in the hospital before ICU admission was associated with improved hospital survival [7]. However, owing to the retrospective nature of this analysis and the possible presence of unknown confounding factors, a recommendation for early admission to the ICU cannot be based on the present data.

Although a low hematocrit value does have a positive influence on the prognosis of ICU patients [35], we identified low hematocrit at ICU admission as an independent risk factor for survival after ICU stay but not for ICU mortality. Patients admitted to the ICU for infectious complications benefit from a restrictive transfusion policy with tolerated hemoglobin values of 7.0 g/dl or below [36]. However, as stated above, our retrospective observation is insufficient to recommend a restrictive transfusion policy with a hematocrit goal of <25%.

ELN low risk was significantly associated with survival after ICU discharge, whereas ELN high risk exhibited only borderline significance. However, in contrast to the intention of ELN risk classification, these cohorts do not represent homogeneous populations of patients with untreated, newly diagnosed disease. The significant influences of disease status and previous allotransplant on prognosis after ICU discharge suggest that ELN risk is diluted by disease status.

Several limitations must be addressed. Our survival index distinguished three separate prognostic groups in the test as well as in the validation cohort, but patient survival was inferior in the validation cohort compared to the training cohort in every risk category. Missing values in the validation cohort (days in hospital before ICU admission, diuresis, and GCS) and imbalances with respect to the proportion of remission status and lower paO2 (see Table 1) are possible explanations, we cannot rule out the possibility that this prognostic index performs worse in independent cohorts than in the training cohort, even with all available variables. Second, only crude paO2 values and not the amount of oxygen support was available at the time of ICU admission. Thus, the paO2:FiO2-ratio (ratio of paO2 to the fraction of inspired oxygen) was not incorporated into the scores. Finally, due to an inverse correlation between hyperleukocytosis and paO2, paO2 values in hyperleukocytic AML may be in spuriously low.[37] Peripheral capillary oxygen saturation (SpO2) might reflect gas exchange in this constellation more accurate.

## Conclusions

Our study indicates promising survival rates for patients with AML requiring intensive care treatment. Based on data from a large multicenter cohort, we identified and validated relevant risk predictors, which provided a basis for two scores distinguishing between survival differences both in the ICU as well as after ICU discharge. However, while these scores might aid the prognostication of patients with AML treated in the ICU, decisions about initiating or pursuing intensive treatment must not rely solely on the results of these scores.

This study should encourage further prospective analyses.

## Supporting Information

S1 Table Overview of all parameters selected for analysis and their classifications. (DOCX)
