# Correction: Patients with Acute Myeloid Leukemia Admitted to Intensive Care Units: Outcome Analysis and Risk Prediction

**DOI:** 10.1371/journal.pone.0190802

**Published:** 2018-01-02

**Authors:** Michele Pohlen, Nils H. Thoennissen, Jan Braess, Johannes Thudium, Christoph Schmid, Matthias Kochanek, Karl-Anton Kreuzer, Pia Lebiedz, Dennis Görlich, Hans U. Gerth, Christian Rohde, Torsten Kessler, Carsten Müller-Tidow, Matthias Stelljes, Carsten Hullerman, Thomas Büchner, Günter Schlimok, Michael Hallek, Johannes Waltenberger, Wolfgang Hiddemann, Wolfgang E. Berdel, Bernhard Heilmeier, Utz Krug

Mr. Carsten Hullermann is not included in the author byline. He should be listed as the fifteenth author and affiliated with Department of Medicine A, Hematology and Oncology, University Hospital Muenster, Muenster, Germany. The contributions of this author are as follows: Data Curation, Validation, Writing–Review & Editing.
