# Supplementary material for: Patients with Acute Myeloid Leukemia Admitted to Intensive Care Units: Outcome Analysis and Risk Prediction
Source: PLoS One. 2016 Aug 30;11(8):e0160871. doi: 10.1371/journal.pone.0160871 (PMC5004890; doi:10.1371/journal.pone.0160871)
Supplement: S1 Table — (DOCX) [file pone.0160871.s001.docx]

**Supplementary Table 1. Classification of variables and results of uni- and multivariate analyses.**

|  | **Classification** | | **Death in ICU** | | **Survival after ICU discharge** | |
| --- | --- | --- | --- | --- | --- | --- |
| ***Variable*** | **0** | **1** | **Univariate** | **Multivariate** | **Univariate** | **Multivariate** |
| **Age** | ≤60 years | >60 years | .511 | n.i. | .296 | n.i. |
| **Sex** | Male | Female | .108 | n.i. | .247 | n.i. |
| **Active disease** | Remission | Newly diagnosed,  relapsed or refractory | .012 | .010 | .681 | n.i. |
| **Advanced disease** | Remission or newly diagnosed AML | Relapsed or refractory | .696 | n.i. | .008 | <.001 |
| **Secondary AML** | No (*de novo*) | Yes (after MDS or therapy-related) | .856 | n.i. | .116 | n.i. |
| **ELN low risk** | No | Yes | .049 | >.05 | .045 | >.05 |
| **ELN high risk** | No | Yes | .832 | n.i. | .054 | >.05 |
| **Previous alloSCT** | No | Yes | .237 | n.i. | .058 | .033 |
| **Severe infection** | No | Yes | <.001 | <.001 | .945 | n.i. |
| **Temperature** | >38°C (100.4°F)  or <36°C (96.8°F) | 36°C to 38°C | .067 | n.i. | .425 | n.i. |
| **Tachycardia** | >90 bpm | ≤90 bpm | .006 | n.i. | .078 | n.i. |
| **Tachypnea** | >20 /min | ≤20 /min | <.001 | n.i. | .627 | n.i. |
| **Microbiological findings** | No | Yes | .702 | n.i. | .730 | n.i. |
| **Time between admission and ICU admission in days** | Continuous variable | | .173 | n.i. | <.001 | .038 |
| **Time in ICU in days** | Continuous variable | | n.e. | n.e. | <.001 | .034 |
| **WBC** | Continuous variable | | .258 | n.i. | .847 | n.i. |
| **Platelets** | Continuous variable | | .124 | n.i. | .209 | n.i. |
| **Hypoxemia** | paO2 ≥72 mmHg | paO2 <72 mmHg | <.001 | <.001 | .680 | n.i. |
| **MAP** | <60 mmHg | ≥60 mmHg | .950 | n.i. | .698 | n.i. |
| **Decreased urine production** | ≥1 l/day | <1 l/day | .001 | >.05 | .032 | .002 |
| **Hemodialysis in ICU** | No | Yes | <.001 | .008 | .493 | n.i. |
| **Decreased Glasgow Coma Scale** | 8 - 15 | ≤7 | .240 | n.i. | .023 | .013 |
| **Mechanical ventilation** | No | Yes | <.001 | <.001 | .743 | n.i. |
| **Decreased hematocrit** | >25% | ≤25% | .604 | n.i. | .006 | .005 |

Variables were classified as “0” or “1” according to the cutoff values. The p values of the uni- and multivariate analyses are stated. Only variables with a p<0.1 value in the univariate analysis were included in the multivariate analysis. Abbreviations: alloSCT, allogeneic hematopoietic stem cell transplantation; AML, acute myeloid leukemia; ELN risk, cytogenetic and molecular genetic risk classification according to the European LeukemiaNet 2010 guidelines (28); ICU, intensive care unit; MAP, mean arterial blood pressure; n.e., not evaluated; n.i., not included; SIRS, systemic inflammatory response syndrome; WBC, white blood count.
